# Salt Treatment Confers Protection Against Acute Carp Edema Virus Reinfection While Promoting Viral Persistence

**DOI:** 10.1111/jfd.70185

**Published:** 2026-04-15

**Authors:** Mikolaj Adamek, Maria Zawisza, Justin Tze Ho Chan, Alexander Rebl, Felix Teitge, Alberto Falco, Anne‐Carina Miebach, Verena Jung‐Schroers, Esteban Soto, Jiří Kyslík, Dieter Steinhagen, Krzysztof Rakus, Tomas Korytar

**Affiliations:** ^1^ Fish Disease Research Unit, Institute for Parasitology University of Veterinary Medicine Hannover Hannover Germany; ^2^ Department of Evolutionary Immunology, Institute of Zoology and Biomedical Research, Faculty of Biology Jagiellonian University Krakow Poland; ^3^ Doctoral School of Exact and Natural Sciences Jagiellonian University Krakow Poland; ^4^ Fish Health Division University of Veterinary Medicine Vienna Austria; ^5^ Institute of Genome Biology Research Institute for Farm Animal Biology (FBN) Dummerstorf Germany; ^6^ Spanish National Research Council (IATS‐CSIC) Institute of Aquaculture Torre de la Sal Cabanes Spain; ^7^ Department of Medicine and Epidemiology, School of Veterinary Medicine University of California Davis California USA; ^8^ Laboratory of Fish Immunology, Biology Centre, Institute of Parasitology Czech Academy of Sciences České Budějovice Czechia

**Keywords:** B‐cells, CEV, KSD, natural immunization, salt treatment

## Abstract

Carp edema virus (CEV) infects the common carp ( *Cyprinus carpio* ) and causes the lethal koi sleepy disease (KSD). Signs of KSD include respiratory, detoxification, and osmoregulatory difficulties. Salt treatment re‐equilibrates blood sodium levels and can save the fish. However, it is unclear whether these fish are immunized, remain chronically infected, and shed the virus, which could cause concern for aquaculture and the international fish trade. To address this issue, we examined the physiological and immunological responses following the infection of naturally immunized and naïve fish. Primary CEV infection induces inflammation in the gills and recruits granulocytic leukocyte infiltrates to the lamellae. Immunologically, the antiviral response driven by type I interferon is activated; however, both T and B lymphocytes fail to respond. As part of an immunization strategy, a primary infection followed by salt treatment effectively nullifies the pro‐inflammatory response and lymphocyte immunosuppression during CEV reinfection. Our data indicate that immunization enables mechanisms such as lymphocyte activation, differentiation, antigen presentation, and an adaptive immune response and antibody production. However, immunized fish are unable to fully clear the virus for a significant period, during which they are most likely to shed infectious particles.

## 1 Introduction

Carp edema virus (CEV) is a highly virulent fish poxvirus that infects the common carp ( *Cyprinus carpio* ) and its ornamental koi varieties. CEV causes a severe gill disease which is called koi sleepy disease (KSD) and can result in mortality of up to 100% of infected fish (Zawisza, Chadzinska, et al. 2024). CEV impairs respiratory, osmoregulatory and excretory function of the gills. It can lead to a decrease in blood oxygenation and an increase in CO_2_ content. However, the most severe physiological symptoms of KSD are significantly reduced sodium levels in the blood plasma (hyponatremia) due to increased ion loss through damaged gills, and elevated ammonia levels (hyperammonemia) due to impaired ammonia secretion (Adamek et al. 2021).

Importantly, CEV has spread all over the world, mostly due to international trade of koi and common carp (Machat et al. 2021; Way et al. 2017). In Germany, the virus has been repeatedly found in shipments of completely asymptomatic fish (Adamek et al. 2016). A very similar occurrence was recorded in shipments of koi in France (Montacq et al. 2025). These virus‐carrying fish were most likely the result of natural immunization performed by fish farmers in Japan. Koi breeders immunize juvenile koi by cohabitation with CEV‐infected fish and then rescuing the fish with a 0.5% NaCl bath combined sometimes with raising water temperature (Miyazaki et al. 2005). Salt treatment is highly effective in preventing most of the morbidity and mortality associated with KSD (Miyazaki et al. 2005; Seno et al. 2003; Stevens et al. 2018). Recently, a successful salt treatment of KSD was described in koi populations in the US, showing its effectiveness in clinical settings and further proof that we can control signs of KSD including the onset of mortality (Stevens et al. 2018).

The salt treatment mechanism is related to balancing the osmotic disturbances and stabilizing the sodium concentration in the blood to its physiological level, thereby reducing mortality due to severe hyponatremia. Salt treatment also reduces ammonia intoxication by facilitating its removal (Adamek et al. 2021; Seno et al. 2003; Zawisza, Rebl, et al. 2024). However, the exact mode of action of salt treatment during KSD is not fully explained yet. Our recent studies showed that salt treatment of CEV‐infected fish resulted in lower stress response (significantly lower cortisol and glucose levels in blood plasma) as compared to non‐salt‐treated CEV‐infected fish (Zawisza, Rebl, et al. 2024). Moreover, in the gills of salt‐treated fish, the CEV‐induced immunomodulation of the host adaptive immune response is limited: expression of selected adaptive immune genes (e.g., *cd4*, *igm*, *tcra1* and *tcra2*) was no longer downregulated in the gills of CEV‐infected salt‐treated fish (Adamek et al. 2021; Zawisza, Rebl, et al. 2024). We hypothesize that the salt treatment can prevent immunosuppression and give fish time to develop adaptive immune responses and better control the virus.

Additionally, despite the clear efficacy of salt treatment, some common carp farmers and ornamental koi breeders reported reoccurring signs related to KSD even after the treatment. After the initial outbreak and salt treatment, their fish sometimes experienced repeated outbreaks of the disease without introducing new fish or reintroducing CEV to the system (Adamek, Heling, et al. 2022). This raises several questions about the ability of the virus to persist in the carp population after salt treatment and about the effectiveness of natural immunity following salt treatment.

This study aims to address the potential advantages and disadvantages of salt treatment and natural immunization, and will provide further indications as to whether immunization and vaccination against CEV can be achieved. The underlying mechanism necessarily implicates host physiology and immunology of the gills, a unique fish mucosal barrier and interface with the external environment that is colonized by its own community of commensal bacteria (Reverter et al. 2018). The combination of case studies documenting the outcome of salt treatments in koi and farmed common carp, as well as experimental infections by cohabitation, were used to answer these timely questions.

## 2 Materials and Methods

### 2.1 Fish

Two groups of common carp (approximately 3–5 g) were bred in separate, well isolated from each other flow‐through outdoor concrete tanks in the Fisheries Department of the Sakson State Agency for Environment, Agriculture and Geology in Koenigswartha (Germany). They were shipped to the Fish Disease Unit, University of Veterinary Medicine in Hannover (Germany) and placed in separate fish rooms in preparation for infection experiment with *cyvirus cyprinidallo3* (CyHV‐3). Shortly upon arrival, one group showed clinical signs of KSD (lethargic behaviour, swollen gills) and was confirmed to be positive for CEV (genogroup I) by means of qPCR analyses, and immediately subjected to 0.5% NaCl treatment for 2 weeks, becoming naturally CEV‐immunized fish. The second group, without any clinical signs and CEV negative (confirmed with multiple samplings and qPCRs), was not salt‐treated and became a control group (CEV naïve fish). In parallel, fish from both groups were also checked by means of qPCR or RT‐qPCR assays for additional specific viruses that infect common carp: CyHV‐3, spring viremia of carp virus (SVCV), and common carp paramyxovirus (CCPV) as described earlier (Adamek et al. 2021). As fish from both groups were disqualified from CyHV‐3 experiment due to involvement of one of the groups with CEV, 2 months after salt treatment, clinically healthy fish from both groups were transported to the Department of Evolutionary Immunology, Institute of Zoology and Biomedical Research, Jagiellonian University in Krakow (Poland) for CEV infection to test their resistance against the virus. Fish from both groups were kept in separate tanks at a water temperature of 18°C, a day/night cycle of 12/12 h, and fed a commercial feed (Perla Plus, Skretting Norway) at a rate of 1% body weight per day.

### 2.2 CEV Challenge and Sample Collection

CEV infection experiments were carried out in accordance with national and international regulations for experimentation with animals, under approval of the 2nd Local Ethics Committee in Krakow, Poland (no. 355/2021). A humane endpoint was set up at a disease stage in which fish were presenting increasing signs of lethargy to the point where the most affected individual showed coma‐like behaviour, lying on the side of the body with visible respiration but without any motor response to prodding with a fishing net.

Before CEV infection, the water temperature was lowered from 18°C to 14°C by 1°C per day and fish were acclimatized to the final water temperatures for 2 weeks. At 3.5 months after NaCl treatment, fish from both groups (naïve and immunized fish) were infected with CEV (genogroup I) by cohabitation with clinically infected, virus‐shedding donor fish (*n* = 3 fish per infected tank) for 24 h. Since propagation of this virus in vitro has not been established yet, donor fish were injected intraperitoneally with a gill homogenate obtained from fish sampled at the beginning of the above‐described outbreak. Infected fish from each group were randomly chosen and placed into 2 tanks: one for survival analysis (*n* = 10 fish per group) and the second one for sample collection (*n* = 15 fish per group). At day 0 (before infection), an additional *n* = 5 fish per group were sampled, followed by a sampling 4‐ and 6‐days post‐infection (dpi). Fish for sample collection were transferred to a tank with an overdose of MS 222 in water (0.4 g/L) and euthanized prior to collection of blood and gills (*n* = 5 fish/group/sampling point). Blood was collected from the caudal vein using S‐Monovette (Sarstedt, Germany) and centrifuged at 2400×g for 10 min at 4°C. Plasma was collected and kept at −80°C for further analysis. Third gill arch on the left side of the body was collected into a 4% formaldehyde phosphate‐buffered solution (Roth, Germany) for gill histopathology, while second gill arch was collected into RNA*later* (Thermo Fisher Scientific, Germany) for DNA/RNA extraction.

### 2.3 CEV Persistence Case Study

A population of 3‐ to 10‐year‐old koi fish bred in our anonymous client's garden pond located in Lower Saxony experienced a mild outbreak of KSD in May/June 2020. In June 2020 (sampling no. 1), 23 individual fish were anaesthetised by MS 222 (150 mg/L) and sampled using swabs for gill tissue. To identify individual fish and follow the course of treatment, we photographed each koi. After confirmation of CEV in gills of sampled fish by qPCR, we transferred fish to a 2000‐L tank for a 12‐day‐long NaCl treatment (0.5%), at a water temperature of 18°C and natural photoperiod. After 12‐day salt treatment, fish returned to healthy appearance; at this point, fish were sampled again (sampling no. 2 in June 2020; at a water temperature of 18°C) using gill swabs and released into the garden pond. Sampling of fish from the garden pond was repeated in October 2020 (sampling no. 3; at a water temperature of 12°C) and June 2021 (sampling no. 4; at a water temperature of 18°C). The presence of virus‐specific DNA in gill swabs was measured each time by qPCR.

### 2.4 Analysis of Sodium and Ammonium Levels in Blood Plasma

Sodium levels were determined with a flame photometer (Bayer Diagnostics, Germany) and ammonium levels were measured by means of a photometric test (LT Sys, Germany) in the blood plasma samples according to the manufacturer's protocol.

### 2.5 Gill Histopathology

From each fish, a gill arch was collected immediately after euthanasia, fixed with a 4% formaldehyde phosphate‐buffered solution and stored at 4°C for 24 h. The samples were dehydrated in a series of graded ethanols and embedded in paraffin wax in accordance with a standard laboratory protocol. Sections (3 μm) of paraffin‐embedded gills were cut and stained with haematoxylin and eosin (HE).

### 2.6 DNA Extraction

DNA was extracted from gill swabs or gill tissue stored in RNA*later*. First, the swabs or tissue were mechanically lysed in a TissueLyser II (Qiagen, Germany), and DNA was extracted using the QIAamp DNA Mini Kit (Qiagen, Germany) according to the manufacturer's protocol. After extraction, DNA was diluted to 50 ng μL^−1^ and stored at −80°C.

### 2.7 Viral Load Analysis

DNA extracted from gill swabs or gill tissue was used as a template for quantification of viral load using a probe‐based qPCR assay (Matras et al. 2017). The gene encoding the P4a capsid core protein of CEV (*p4a*) was used as a target. The reaction mix contained 1× Maxima Probe qPCR Mastermix (Thermo Fisher Scientific, Germany), 500 nM of each primer, 200 nM of the probe, 3 μL of template DNA and nuclease‐free water to a final volume of 10 μL. The reaction was performed in a StepOnePlus thermocycler (Applied Biosystems, Germany). The amplification program included an initial denaturation at 95°C for 10 min, followed by 40 cycles of denaturation at 95°C for 30 s and annealing at 60°C for 30 s. A standard curve from 10^1^ to 10^7^ copies of the *p4a* gene fragment was used for quantification of the copy number of CEV‐specific DNA in each sample. The results for virus load are presented as the number of virus‐specific DNA copies per 250 ng of total DNA.

### 2.8 Gene Expression Analysis

Gene expression was studied in gills using multiplex RT‐qPCR. The target genes of B cell markers of activation, proliferation, and differentiation were selected based on a panel of markers of various stages of B cell differentiation as identified by single‐cell RNA sequencing of head kidney grass carp IgM^+^ B cells (Pan et al. 2023) and combined with other immune responses markers (Adamek, Matras, et al. 2022). The combination of RT‐qPCR and integrated fluidic circuit (IFC) technology of Standard BioTools was used to profile the expression of target genes and 4 reference genes (Table S1). For these multiplex RT‐qPCR analyses, 48.48 Gene Expression IFC chips (Standard BioTools) were used with the BioMark HD system (Standard BioTools) as described earlier (Adamek, Matras, et al. 2022). Results were analysed with the Standard BioTools RealTime PCR Analysis Software v.4.5.2 and normalized against the geometric mean of the reference genes *actb, gapdh, eef1a1*, and *rps11*. Heatmaps of gene expression were created with Heatmapper (Babicki et al. 2016).

### 2.9 Bacterial Analysis in Gills

Quantification of bacteria was performed using qPCR as described earlier (Adamek, Matras, et al. 2022). Assays for all bacteria, Aeromonas, Flavobacteria and Pseudomonas 16S were used to assess the stability of the bacteria community during CEV challenge.

### 2.10 Statistical Analysis

Statistical analysis was carried out in the SigmaPlot 12.5 software (Systat Software). Data from viral and bacterial load and gene expression studies were transformed using a Log10(x) transformation before statistical analysis. Significant differences (*p* ≤ 0.05) were assessed using a two‐way ANOVA, with subsequent pairwise multiple comparisons using the Holm–Sidak test.

## 3 Results

### 3.1 Confirmation of CEV Infection in Fish

One group of fish which were imported from the Fisheries Department of the Sakson State Agency for Environment, Agricultural and Geology in Koenigswartha (Germany) showed clinical signs of KSD upon arrival the Fish Disease Unit, University of Veterinary Medicine in Hannover (Germany). They were confirmed to be CEV‐positive (mean of 1.35 × 10^5^ copies of virus‐specific DNA per 250 ng of total DNA), second genogroup II selective qPCR, confirmed that fish were infected with CEV from genogroup I, while Sanger sequencing of the P4a gene fragment confirmed an identical sequence to that previously detected in Saxony (e.g., GenBank ID: OP494134). Fish from the second group bred in other tank and showing no clinical signs of KSD were CEV‐negative across samplings performed at arrival, 1 month of keeping and prior transport to the Department of Evolutionary Immunology, Institute of Zoology and Biomedical Research, Jagiellonian University in Krakow (Poland). Fish from both groups were negative for other viruses tested (CyHV‐3, SVCV, and CCPV).

### 3.2 Natural Immunization With CEV Protected Fish Against Reinfection With the Virus

**FIGURE 1:**
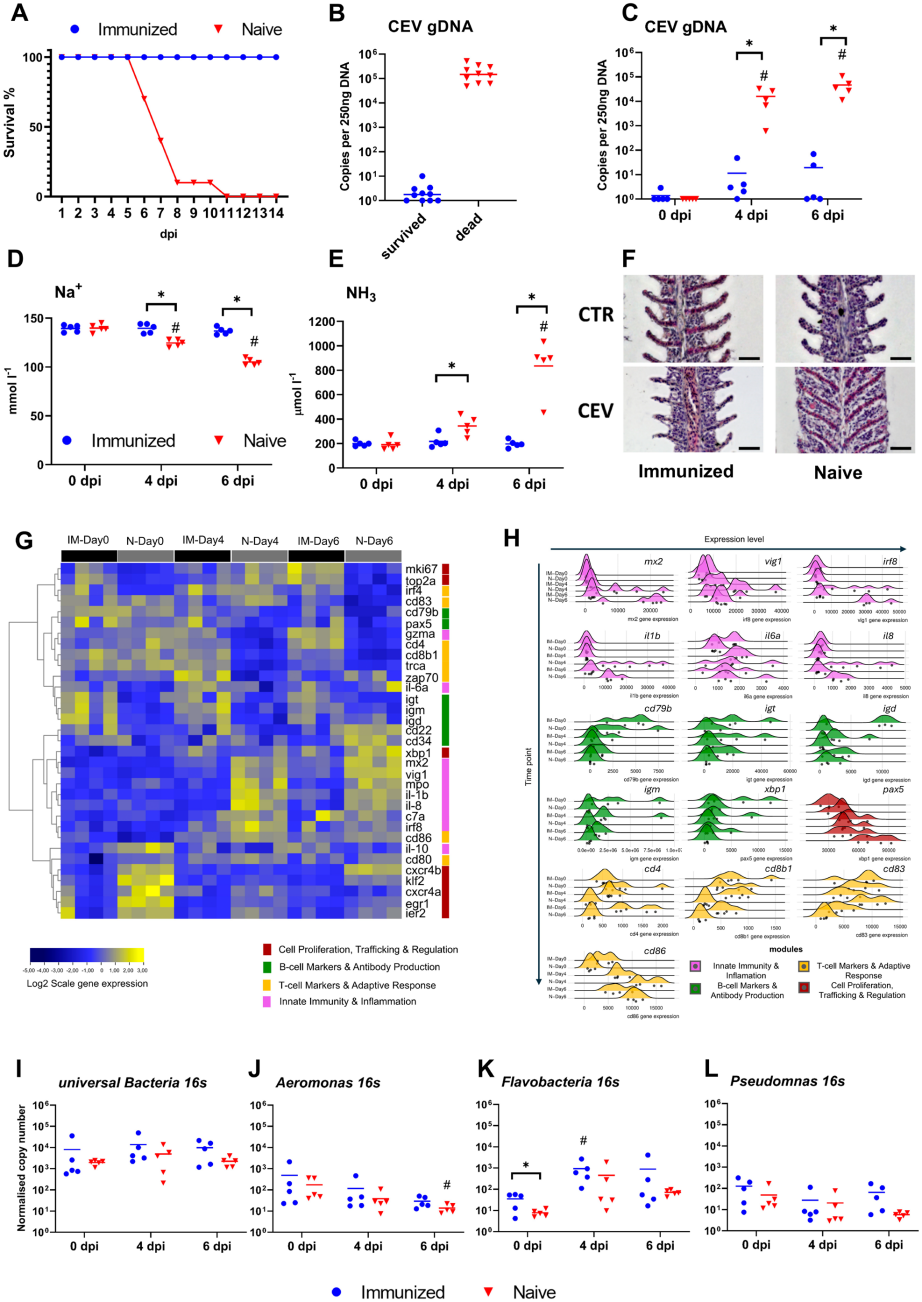
(A‐F). **Viral load, physiological and histopathological changes following salt treatment and CEV (re)infection**. Koi carp were either immunized prior via CEV infection and salt treatment rescue (Immunized), or had never been CEV‐infected nor salt‐treated (Naïve). We infected these two groups of fish by cohabitation with CEV‐positive donors. (A) The survival curve depicts the percentage of surviving fish following re‐exposure for the immunized group, or primary exposure for the Naïve group. (B) Endpoint (14 dpi) absolute quantification of the number of copies of CEV genomic DNA (gDNA) in the gills of surviving fish in the former group, and deceased fish (at humane endpoint i.e., 6‐11dpi) in the latter group. We followed the course of infection from immediately prior to infection (0 days post‐infection), 4 dpi up to the 6 dpi timepoint. Whereas Immunized fish were unchanged by every metric, the Naïve group viral load (C) was inversely correlated with their serum sodium concentration (D), and correlated with rising levels of serum ammonia (E). (F) Representative haematoxylin‐ and eosin‐stained gill filaments from the Immunized and Naïve groups immediately prior to infection (CTR) or at its endpoint 6 dpi (CEV), black scale bar = 50 μm. *n* = 5 fish per group. **Gill expression profiling of inflammatory, innate immune, and lymphocyte markers following CEV infection** (G and H). Immunized and salt bath‐rescued koi carp ('IM' = Immunized) or non‐immunized non‐treated ('N' = Naïve) fish were infected with CEV. Prior to (0dpi) or up to 6 days dpi, we biopsied gills to profile expression of type I IFN‐stimulated genes (*mx2* and *vig1*), complement component *c7a*, inflammatory markers (*il1b*, *il6a*, and *il8*), the regulatory cytokine *il10*, T cell markers (*gzma, zap70*, *cd4*, *cd8b1*, and *trca*), and neutrophil *mpo*. We selected also a panel of common carp orthologues of markers initially identified by single‐cell RNA sequencing of grass carp head kidney IgM^+^ B cells. *n* = 4 fish per group. (I,J,K and L) **changes in gill microflora in immunized (Immunized) and non‐immunized (Naïve) fish following CEV infection**. We measured changes in total bacterial load as well as that of three genera during CEV infection. Data are in normalized copies of 16S rDNA of *Flavobacterium* spp., *Pseudomonas* spp., and *Aeromonas* spp. *n* = 5 fish per group. *Indicates the statistically significant difference (at *p* ≤ 0.05) between groups at ach timepoint, # indicates the statistically significant difference (at *p* ≤ 0.05) between 0dpi and other time points.

Fish that were CEV‐positive and subjected to NaCl treatment (naturally immunized fish), and fish that were negative for CEV (naïve) were cohabitated with CEV‐shedding donor fish 3.5 months post‐salt treatment to mimic acute (re)infection with CEV. After (re)infection with CEV, immunized fish showed 100% survival whereas CEV‐infected naïve fish were euthanized when reaching the humane endpoint with the approximated mortality peak between 5 and 8 dpi (Figure 1A). Quantification of CEV gDNA by qPCR in gills of immunized fish that survived the infection (sample collected at 14 dpi) showed a very low viral load (below 10 copies of virus‐specific DNA per 250 ng of total DNA) (Figure 1B). In naïve fish that were euthanized after reaching endpoint, the viral load was signifcantly higher (mean over 10^5^ copies of virus‐specific DNA per 250 ng of total DNA) (Figure 1B).

Kinetics of CEV infection of immunized and non‐immunized naïve fish were also quantified in gills up to 6 dpi (when naïve fish started to reach the humane endpoint due to KSD) (Figure 1C). In the immunized group, only a small portion of fish were positive for CEV, and the viral load was low at every sampling point (4 and 6 dpi) (mean below 10^2^ copies of virus specific DNA per 250 ng of total DNA). In the naïve group, all fish were positive for CEV and increased viral load was observed during the experiment with a mean of approximately 10^4^, and 5 × 10^5^ copies of virus‐specific DNA per 250 ng of total DNA at 4 and 6 dpi, respectively (Figure 1C). The highest viral load observed at 4 and 6 dpi correlated with significantly decreased sodium levels and significantly increased ammonia levels in the blood plasma of CEV‐infected non‐immunized fish (Figure 1D,E). No significant changes in sodium and ammonium levels in the blood plasma were observed in immunized fish post‐CEV exposure (Figure 1D,E).

Gill tissues collected at 6 dpi were studied for histopathological changes. Before any CEV re‐infection, we note that the immunized/salt‐bathed fish have secondary lamellae that are more strongly eosin‐staining in contrast to the completely naïve and non‐treated group (Figure 1F, top row). CEV‐reinfected immunized/salt‐treated fish maintained distinguishable, separate, albeit potentially hypoplasic shortened secondary lamellae (Figure 1F, bottom left image). In this group we can find eosinophilic cell infiltrates in the primary filament/lamella that are not seen in any other condition or timepoint. In contrast, CEV infection of non‐immunized fish fused the secondary lamellae, potentially due to inflammation and hyperplasia. These lamellae were also more eosinophilic (Figure 1F, bottom right image).

### 3.3 Differential Expression of Immune‐Related Genes Between Naturally Immunized and Naïve Fish During CEV Infection

To better understand the mechanisms underlying the divergent outcomes of CEV infection, we profiled immune gene expression in the gills of naïve and naturally immunized fish across the early stages of infection (0, 4, and 6 dpi). To this end, we employed a panel of prototypical markers for inflammation, cell recruitment, and development of both T and B cell responses (Figure 1G,H and Figure S1).

At baseline (0 dpi), there was no indication of immune priming from either group when analysing innate immune, antiviral, and inflammatory gene transcription (Figure 1G). However, immunized fish expressed markedly higher levels of *gzma, cd4, igm*, *igd*, *irf4*, *mki67* and *top2a*, suggesting the heightened presence of memory, effector, or antibody‐secreting cells (Figure 1G). In contrast, naïve fish showed elevated expression of *cxcr4a, cxcr4b*, and early response transcription factors *egr1* and *ier2*, potentially reflecting a more reactive, uncommitted immune landscape.

Upon infection, naïve fish mounted a strong but ultimately insufficient immune response, marked by a significant upregulation of interferon‐stimulated genes (*mx2, vig1*), pro‐inflammatory cytokines (*il‐1β, il‐6a, il‐8*), and myeloperoxidase (*mpo*), indicative of innate immune activation and myeloid cell recruitment at 4 dpi (Figure 1G). Expression of complement component *c7a* was also increased, suggesting complement system engagement. Despite this activation, expression of T cell markers—including *cd4, cd8b1, zap70*, and *tcra*—was progressively downregulated, likely due to tissue damage, infiltration of myeloid cells or lymphocyte attrition (Figure 1H). The downregulation of both T and B cell signatures was further exacerbated at 6 dpi, coinciding with high viral loads and extensive gill pathology (Figure 1), suggesting that inflammation and innate immune activation failed to prevent viral dissemination and led to immunopathology.

In contrast, immunized fish displayed a restrained and focused response, with most gene expression levels remaining stable throughout the infection. Modest upregulation of *gzma, zap70, cd4*, and *mx2* was observed at select timepoints, possibly reflecting recall activation of cytotoxic and helper T cells (Figure 1G). However, key pro‐inflammatory genes, including *il‐1β, IL‐6a, IL‐8*, and *MPO*, remained unchanged, consistent with the absence of histopathological inflammation and myeloid infiltration. Expression of *xbp1* and *mki67* remained elevated, indicative of reactivation or antibody production from memory B cells or plasma cells (Figure 1G). Immunoglobulin transcript levels (*igm*, *igd*, *igt*) remained relatively stable, suggesting sustained antibody production, although some downregulation was observed at 6 dpi, possibly reflecting feedback inhibition following neutralization of the virus (Figure 1H).

### 3.4 Change in Bacterial Microflora in Naturally Immunized and Non‐Immunized Fish During CEV Infection

As host immunity maintains the mucosal barrier and homeostasis, we hypothesized that if they break down, it would cause an imbalance of the bacterial microflora and dysbiosis, in a vicious cycle. CEV infection did not induce changes in the total bacterial load during infection in both groups of fish (Figure 1I). However, we analysed the bacterial load of three genera: *Aeromonas* spp. (Figure 1J), *Flavobacterium* spp. (Figure 1K), and *Pseudomonas* spp. (Figure 1L). The gills of immunized fish (4 dpi) demonstrated increased loads of *Flavobacterium* spp. The gills of non‐immunized fish contained a decreased load of *Aeromonas* spp. (6 dpi). Moreover, at day 0, naturally immunized fish showed higher *Flavobacterium* spp. load as compared to naïve fish. No changes in the load of *Pseudomonas* spp. were observed.

### 3.5 Persistence of CEV After NaCl Treatment

Koi fish kept in a small garden pond experienced a mild outbreak of KSD in May/June 2020. Viral load analysis showed that all tested fish (sampling no. 1; *n* = 23) were positive for CEV genogroup IIa with a virus load ranging from 5 to 1600 copies of virus‐specific DNA per 250 ng of total DNA. After a 12‐day‐long NaCl treatment (0.5%), the clinical signs subsided and only one fish died (Table 1). Despite being free of clinical signs, sampling no. 2 revealed that 14 of 22 fish remained positive for CEV with a virus load from 3 to 160 copies of virus‐specific DNA per 250 ng of total DNA. During a routine autumn health check in October 2020 (sampling no. 3), three of 11 fish remained CEV‐positive with a similar virus load but still free of clinical signs. A further check‐up performed 1 year after the outbreak (sampling no. 4) showed that all fish were negative for CEV (Table 1). By following a small population of fish in a pond, we show that some individuals were long‐term carriers of CEV and that the virus persists in fish for at least 4 months.

**TABLE 1:** The virus loads during CEV persistence case study.

Sample	Virus load–05/06.2020–initial screening		Virus load–06.2020–after salt treatment		Virus load–10.2020–autumn check		Virus load–06.2021–1 year check	
Fish	Cт mean	Quantity mean	Cт mean	Quantity mean	Cт mean	Quantity mean	Cт mean	Quantity mean
F 01	37.0	5.9	35.0	11.8	No detection	No detection	No detection	No detection
F 02	30.9	254.6	No detection	No detection	No sample	No sample	No detection	No detection
F 03	28.0	1600.7	No detection	No detection	No detection	No detection	No detection	No detection
F 04	31.3	203.8	35.2	10.0	No detection	No detection	No detection	No detection
F 05	32.4	102.0	No detection	No detection	No sample	No sample	No detection	No detection
F 06	34.2	34.0	No detection	No detection	No sample	No sample	No detection	No detection
F 07	33.7	45.8	36.0	6.0	No sample	No sample	No detection	No detection
F 08	33.5	53.3	33.7	28.8	No sample	No sample	No detection	No detection
F 09	33.6	47.5	34.3	19.1	34.3	18.9	No detection	No detection
F 10	34.3	31.4	37.1	2.8	30.6	251.2	No detection	No detection
F 11	34.1	35.4	No detection	No detection	No detection	No detection	No detection	No detection
F 12	29.4	654.1	32.6	57.4	No sample	No sample	No detection	No detection
F 13	28.9	924.2	34.4	18.1	No sample	No sample	No detection	No detection
F 14	31.0	243.1	37.3	2.5	No sample	No sample	No detection	No detection
F 15	30.9	264.4	34.7	15.1	No detection	No detection	No detection	No detection
F 16	29.3	704.5	No detection	No detection	No sample	No sample	No detection	No detection
F 17	33.1	67.4	No detection	No detection	No detection	No detection	No detection	No detection
F 18	30.5	345.8	No detection	No detection	No detection	No detection	No detection	No detection
F 19	31.1	233.2	35.3	9.6	35.0	12.1	No detection	No detection
F 20	29.4	677.9	32.9	48.9	No detection	No detection	No detection	No detection
F 21	29.0	847.6	31.0	183.2	No sample	No sample	No detection	No detection
F 22	29.0	845.4	No sample^a^	No sample^a^	—	—	—	—
F 23	30.6	319.0	34.8	16.8	No sample	No sample	No detection	No detection

^a^ Fish died.

## 4 Discussion

Until recently, only a few fish poxviruses had been described. Among these, CEV and salmon gill poxvirus (SGPV) are highly detrimental to the aquaculture industry (Zawisza, Chadzinska, et al. 2024). These viruses cause widespread diseases in important cultured fish species, such as the common carp and Atlantic salmon ( *Salmo salar* ) (Gjessing et al. 2018, 2017; MacNeill et al. 2025; Matějíčková et al. 2020). Following the recent detection of poxviruses in black bullhead ( *Ameiurus melas* ) (Abonyi et al. 2025) and Atlantic cod ( *adus morhua* ) (Gjessing et al. 2025), it is evident that the number of pathogenic poxviruses affecting fish is growing rapidly. This requires significantly more effort to understand the biology of these pathogens and prevent their spread. However, working with these viruses is extremely difficult as in vitro cell culture methods for replication are unavailable despite numerous attempts at development over many years (Takafumi Ito, personal communication; (Felten et al. 2022; Slattery et al. 2023)). Current infection models largely rely on cohabitation with virus carriers, which must be obtained from the field (Adamek et al. 2021; Solhaug et al. 2023). This leads to significant limitations in the available experimental designs and makes any effort to understand virus biology and fish immunity much more valuable even if they are opportunistic, with not fully controlled experimental design (Thoen et al. 2020).

Poxvirus infections in warm‐blooded animals are characterized by an acute infection, whey replicate in cytoplasm therefore there is no possibility for latency, furthermore there is no clear evidence of persistent or chronic infections in most cases (Buller and Palumbo 1991). However, there are exceptions, such as the rabbit fibroma virus (RFV), which causes cutaneous fibroma and the infection can persist for months (Barrett and McFadden 2007). On the other hand, some poxviruses like avian pox or vaccinia virus can persist in the environment and reinfect their hosts when conditions are favourable (Mei et al. 2024; van Riper III and Forrester 2007). Here, we provide clear evidence that CEV targets and persists in the gills of infected fish for at least 4 months and can be detected in swabs sampling gill epithelium. Although there is potential for CEV persistence for an even longer period than was observed in the present study. This may be due to limitations in number of sampling points and detection limits between gill swabs and gill tissue, as this method has not yet been fully tested for CEV. High potential of long persistence can explain the very successful spread of the virus by the worldwide trade of common carp and koi (Way et al. 2017). Without proper screening procedures and diagnosis, subclinically infected fish can be the source of KSD outbreaks when imported to new locations. This was already demonstrated by the screening of fish shipped to Germany or France, where multiple fish batches were CEV‐positive without any clinical signs of KSD (Adamek et al. 2016; Montacq et al. 2025). Long persistence would also explain why farmers observing new CEV infections without introducing new individuals to a population (Adamek, Heling, et al. 2022).

The absence of in vitro culture systems for piscine poxvirus constrains the development of therapeutic strategies and vaccines (Amundsen et al. 2025). For CEV, as part of an immunization strategy, salt treatment can be used to stop the CEV‐induced mortality (Miyazaki et al. 2005; Seno et al. 2003; Stevens et al. 2018). To explore the persistence and the effect of natural immunization, we followed a group of koi carp during an outbreak of KSD. We also reinfected a group of common carp treated with salt after a natural outbreak of KSD (Figure 1). We demonstrated that fish that were immunized by natural infection with CEV and then rescued with salt treatment were protected against reinfection with the virus for at least three and a half months. Crucially, the gills remained intact, fish blood composition was normal, and leukocytes did not react or potentially overreact to the secondary infection. To understand the pathogenesis of CEV or protection against the virus, we studied physiology, immunology, and the microbiome, which all intersect in the gills (Adamek et al. 2021; Wood and Eom 2021; Zawisza, Chadzinska, et al. 2024).

In the most severe cases of KSD, gill function was compromised as indicated by increased serum ammonia and reduced sodium levels. The pathological changes in the gills, such as fusion of the lamellae and hyperplasia, greatly reduce the surface area, osmoregulatory and excretory capacity of the gills (Pikula et al. 2021; Zawisza et al. 2025). Our data indicate that inflammation may drive this phenomenon by—for example, recruiting inflammatory neutrophils via IL‐8 (de Oliveira et al. 2013; Havixbeck and Barreda 2015; Progatzky et al. 2016; van der Aa et al. 2010). They may be the reason for eosin‐staining and granule‐rich infiltrates in the gills during the CEV infection and can be related to activation of acute phase responses (Machat et al. 2024). It is unclear if they are responding to dysbiosis (which was not clearly present in current studies), viral infection, and/or inflammation (Zawisza et al. 2026), because these cells are also present in immunized fish where all of the above are relatively under control. The increased baseline levels of *il‐10* may help prevent runaway inflammation, promote repair, and maintain gill integrity (Bottiglione et al. 2020).

From an immunological perspective, the fish gill is a mucosa‐associated lymphoid tissue (Dalum et al. 2015; Haugarvoll et al. 2008). Specifically, it is an inductive site where presumably immune responses are mounted, but also an effector site where leukocytes maintain gill homeostasis and the barrier with the external environment (Aas et al. 2017). Thus, it is susceptible to stress (e.g., cortisol), to infection, and to immunomodulation (Zawisza, Rebl, et al. 2024). The gill lymphoid tissue is populated by both CD4^+^ and CD8^+^ cytotoxic T cells (Dalum et al. 2015), the latter partly specializing in antiviral immunity, suggesting they are important in mucosal homeostasis (Takizawa et al. 2011) and protection against CEV. Our previous study demonstrated that CEV modulates the host's immune response by downregulating the expression of adaptive immune genes (Adamek et al. 2021; Zawisza, Rebl, et al. 2024). The transcriptional trajectories observed during the primary CEV infection in naïve fish are in accord with these findings, suggesting polarization into the inflammatory/antiviral axis coupled to a loss of T cell signatures. This may explain the ineffective antiviral response compared to immunized fish, where expression of all these markers is intact and the fish are protected from reinfection. Thus, these findings elaborate on prior observations that CEV suppresses adaptive immune responses (Adamek et al. 2021; Zawisza, Rebl, et al. 2024) and align with the broader principle demonstrated in poxvirus models, where CD4‐dependent help is crucial for effective viral clearance, including the licensing of cytotoxic responses (Harbour et al. 2023). In contrast, naturally immunized fish exhibit an anticipatory, non‐inflammatory protective signature, driven by increases in *gzma*, *zap70* and *cd4*, consistent with CD8‐mediated cytotoxic responses, suggesting viral neutralization and clearance without collateral inflammation.

Since one goal of vaccination/immunization is to confer immunological memory mediated by memory and plasma cells, we also examined the B cell contribution to primary and secondary responses using a panel of B cell lineage activation and differentiation markers published previously (Chan et al. 2024; Pan et al. 2023). Globally, our data suggest activation of antibody responses in both primary (naïve group) and secondary (immunized group) infections, although it follows different trajectories: while immunized fish exhibited increased levels of immunoglobulin transcripts coupled with sustained *xbp1* and *irf4* expression, suggesting constitutive antibody secretion at the gill surface; in naïve fish, rise of these transcripts appeared only within a strong inflammatory milieu and coincided with reduced expression of *cd79b*. At later timepoints, naïve gills exhibited a dysregulated B cell response, characterized by the loss of the B cell signature (*cd79b*) and inconsistent activation, without a proportional increase in immunoglobulin transcripts, leading consequently to the exhaustion of B cells. In contrast, the immunized fish exhibit a stable recall response, characterized by an increase in *xbp1*, *mki67*, or *top2a*, and sustained immunoglobulin transcripts, providing protection against viral replication. Despite not having uncovered precise targetable B cell populations, the global trends and response in the gills suggest that the humoral/antibody response is protective. It can be interrupted by barrier breakdown, stress, and, ultimately, immunosuppression (Zawisza, Rebl, et al. 2024), whereas immunization may maintain mechanisms such as lymphocyte activation, proliferation, chemotaxis, and antigen presentation that are key to long‐term protection (Chan et al. 2024).

Overall, we were able to show the pathophysiology, immunology and virology of KSD are tightly intertwined, despite our opportunistic experimental setup. Therefore, in CEV infection, the sequence of events is not fully elucidated, but it potentially self‐perpetuates if the virus compromises the mucosal barrier (Adamek et al. 2021). This leads to inflammation, immunosuppression of lymphocytes, toxicity and further barrier dysfunction in a vicious cycle (Zawisza, Rebl, et al. 2024). Here, we indicate that salt treatment may not only be therapeutic but extrapolating from the current results, we can also suggest that it may have a prophylactic effect, enabling the development of immunological memory and protection. The interaction between the gill, environment and poxviral infections poses unique challenges. The long persistence of CEV in the gills poses a clear biosecurity challenge and may explain its successful global spread. Conversely, the protection against reinfection provided by increased T cell and B cell responses offers basis for the development of effective CEV vaccines.

## Author Contributions

M.A.: Conceptualization, methodology, data curation, validation, supervision, funding acquisition, visualization, writing – original draft, writing – review and editing. M.Z.: Methodology, investigation, writing – review and editing, writing – original draft. J.T.H.C.: Methodology, investigation, writing – original draft, writing – review and editing. A.R.: Methodology, data curation, investigation, writing – review and editing. F.T.: Methodology, investigation, writing – review and editing. A.F.: Data curation, investigation, writing – review and editing. A.‐C.M.: Methodology, investigation, writing – review and editing. V.J.‐S.: Methodology, data curation, writing – review and editing. E.S.: Methodology, data curation, writing – review and editing. J.K.: Data curation, investigation, visualization, writing – review and editing. D.S.: Methodology, supervision, writing – review and editing. K.R.: Conceptualization, writing – original draft, writing – review and editing, data curation, supervision, funding acquisition, visualization, investigation. T.K.: Conceptualization, data curation, investigation, visualization, supervision, writing – original draft, writing – review and editing, funding acquisition.

## Funding

This work was supported by Deutsche Forschungsgemeinschaft, 426513195. Narodowe Centrum Nauki, UMO‐2018/31/F/NZ6/02311. European Commission, 101084204 Cure4Aqua.

## Conflicts of Interest

The authors declare no conflicts of interest.

## Supporting information


**Figure S1:** Gill expression profiling of inflammatory, innate immune, and lymphocyte markers following CEV infection (G and H). Immunized and salt bath‐rescued koi carp (Immunized) or non‐immunized non‐treated (Naïve) fish were infected with CEV. Prior to (0dpi) or up to 6 days dpi, we biopsied gills to profile expression of type I IFN‐stimulated genes (*mx2* and *vig1*), complement component *c7a*, inflammatory markers (*il1b*, *il6a*, and *il8*), the regulatory cytokine *il10*, T cell markers (*gzma, zap70*, *cd4*, *cd8b1*, and *trca*), and neutrophil *mpo*. We selected also a panel of common carp orthologues of markers initially identified by single‐cell RNA sequencing of grass carp head kidney IgM^+^ B cells. *n* = 4 fish per group. * indicates the statistically significant difference (at *p* ≤ 0.05) between groups at ach timepoint, # indicates the statistically significant difference (at *p* ≤ 0.05) between 0dpi and other time points.
**Table S1:** Primers used in this study.

## Data Availability

The data that support the findings of this study are available from the corresponding author upon reasonable request.
