# Supplementary material for: Salt Treatment Confers Protection Against Acute Carp Edema Virus Reinfection While Promoting Viral Persistence
Source: J Fish Dis. 2026 Apr 15;49(9):e70185. doi: 10.1111/jfd.70185 (PMC13432179; doi:10.1111/jfd.70185)

**Supplementary Table 1:** Primers used in this study.

| **Gene symbol** | **NCBI accession code** | **Gene product** | **Marker specificity** | **Sense and antisense primer (5´-3´)** | **Primer efficiency [%]** | **Amplicon length [bp]** |
| --- | --- | --- | --- | --- | --- | --- |
| *actb* | XM_019089432 | cytoplasmic actin 1 | all cells | GCATCAGGGAGTGATGGTT  GGCCTCATCTCCCACATAG | (Reference gene) | 58 |
| *c7a* | XM_019063856 | complement C7a | complement | ATCTGCCAAAACGCACAATC  GGCTGCTCACACCAAATACA | 98.60 | 120 |
| *cd4* | DQ400124 | CD4 molecule | T helpers | ACCGAGAGTGAAGGTAGAAGAAA  TTCACGTCTTTCGCCCTTTGGA | 100.60 | 123 |
| *cd8b1* | EU025120 | CD8a1 molecule | cytotoxic T cells | GGCTTCTATTCCTGCATGTTTA  AGGATTCACTCCAGGCATTAT | 86.70 | 75 |
| *cd22* (*LOC109081467, LOC109081648*) | XM_042754392, [XM_042726754](https://www.ncbi.nlm.nih.gov/nucleotide/XM_042726754.1?report=genbank&log$=nuclalign&blast_rank=10&RID=4C3RXRTW016) | cluster of differentiation 22 | CD22^+^ cells | CATTCTCTTGCTGATGTCCTTCAT,  ATGAGCGTGTGTTCAGAGGAGA | 88.48 | 99 |
| *cd34* | XM_042713470 | cluster of differentiation 34 | CD34^+^ cells | CTTCACAGTTGCTGGGGACAG,  AACAGGTTCAAAAGCAGGTCCAA | 85.39 | 109 |
| *cd79b* | XM_042767177 | cluster of differentiation 79b | naїve B cells | CCTCTCAGCTGAAGTAAATATCC,  TGACATCTGATCCATCTTTGACG | 92.74 | 109 |
| *cd80*  (*LOC109045134*) | XM_042740109 | cluster of differentiation 80 | proliferating B cells | CACAGCTGAGTACAGCGTTCC,  AGGTGGACGGGGCTTAATCAAA | 95.26 | 143 |
| *cd83*  (*LOC109106145*) | XM_019119505 | cluster of differentiation 83 | proliferating B cells | CCGTCATATGGTACAAGGTTTCT,  TGTCTAATGTAACTGCAGAGGAC | 87.66 | 173 |
| *cd86*  (*LOC109076482*) | XM_042778525 | cluster of differentiation 86 | proliferating B cells | GTGCGCCATTCTTCATTAAGGG, CTACATGAGGACGTCAGACTAG | 92.14 | 148 |
| *cxcr4a* | XM_042725575 | C-X-C chemokine receptor type 4 alpha | proliferating B cells | TACGAACACATCGTCTTTGAAGAT, GAGCCAACTTTGAGGTTCCGTG | 94.77 | 92 |
| *cxcr4b*  (*LOC109070166*) | XM_042763477 | C-X-C chemokine receptor type 4 beta | proliferating B cells | CTTCTTCATCTGTTGGTTGCCTTA,  TGCTGTCTCAACCCCATCCTTTA | 93.73 | 165 |
| *eef1a1* | AF485331 | elongation factor 1-alpha 1 | all cells | ATTGGAGGTATTGGAACCGT  CAAAGGTCACAACCATACCTG | (Reference gene) | 76 |
| *egr1* | XM_019064348 | early growth response protein1 | proliferating B cells | ACTGGAGACACGCTTTCAGAAAT,  TCTTACACGGGCCGTTTCACC | 97.00 | 102 |
| *gapdh* | AJ870982 | glyceraldehyde-3-phosphate dehydrogenase | all cells | GCAAGCTGGTCATTGACG  GTTGGCTGGATCCCTCTC | (Reference gene) | 59 |
| *gzma* | GU362096 | granzyme A/K | T and NK cells | GTGTTGGCATCGTCAGTTACG  AGTACCCCAACCTGTCACG | 105.15 | 265 |
| *ier2*  (*LOC109049619*) | XM_019067280 | immediate early response protein 2 | proliferating B cells | CTCTAGAAAGCGACGGAGCAAA,  CGTGCCTATGCCAAGAACAATTG | 94.13 | 173 |
| *ighd*  *(LOC109106335)* | XM_042732961 | immunoglobulin D heavy chain (predicted) | IgD-positive B cells | GGTCACTGTCTCGTCAGTTCAA  TCACCATCGGCTGCTTGGCAA | 99.96 | 104 |
| *ighm* | [AB004106](https://www.ncbi.nlm.nih.gov/nucleotide/AB004106.1?report=genbank&log$=nuclalign&blast_rank=4&RID=4C5G5EWR013), AB004107, MH352354, MH352353 | immunoglobulin M heavy chain membrane-bound and secretory protein | IgM-positive B cells | CGAATATGCAGTTCCTATTCAAGAT  AACATGTGGAACTTGATGCCCC | 97.55 | 216 |
| *ight* | AB598368 | Immunoglobulin heavy chain T2 | IgT-positive B cells | GAAGGATACAAATGCTCCGTTAC  ATAACAAGTGAGCAACGGGACAA | 98.60 | 133 |
| *il1b* | AJ245635 | Interleukin-1 beta | pro-inflammatory | CCCAGATCAACTAGGCATGATG  TTCAACTTTTCCACAGCGATGAC | 94.30 | 123 |
| *il6a* | KC858890 | interleukin 6a | pro-inflammatory | CAGATAGCGGACGGAGGGGC  GCGGGTCTCTTCGTGTCTT | 93.50 | 190 |
| *il8 (cxcl8)* | AB470924 | interleukin 8 | pro-inflammatory | ATCCACGCTGTCGCTGCATT  TGCAGTAGGGTCCAGACAGA | 101.60 | 134 |
| *il10* | AB110780 | interleukin 10 | anti-inflammatory | ACTTGGAACCATTATTGAATGAA  GACGTTACATCCATAAGGACTATTG | 99.70 | 65 |
| *irf4*  (*LOC109113750*) | XM_019126566 | interferon regulatory factor 4 | innate B cells | GGCCCTCTCAGATTACCGCTTA,  TAGTCCAGAGGGCTGTCAGCT | 103.31 | 93 |
| *irf8*  (*LOC109049429, LOC109078735*) | XM_042754085, XM_042743728 | interferon regulatory factor 8 | innate B cells | ATCTTCAAAGCGTGGGCGATATT,  ATTTTGAGGAAGTTACTGACCGAT | 91.43 | 130 |
| *klf2*  (*LOC109047782*) | XM_019065449 | Krüppel like factor 2 alpha | proliferating B cells | GAAAACAGGTGGAAGGAGGAAC, TTATTCTGGCCAACACTGTGGG | 86.07 | 146 |
| *mki67* | XM_042735109 | marker of proliferation Ki-67 | proliferating B cells | GTTCGGAAGGAAGCTGGACTG, AGAACAAGGAGCTCATTTTGACC | 100.37 | 103 |
| *mpo* | AB429306 | myeloid-specific peroxidase | neutrophils | TACCAGGCTATAATGCATGGCG  GCGATGCCTCCCAACCAAATG | 100.0 | 157 |
| *mx2* | XM_019081222 | MX dynamin like GTPase 2 | antiviral | AGGGAAGAGTTCTGTTCTCGA  GGCATCTTGTAACAATTCCACTA | 97.70 | 74 |
| *pax5* | XM_019109360 | paired box 5 | naїve B cells | CAACAGGATCATTCGCACTAAAG, GTGACCCAGGTATCTGCAGTAA | 87.60 | 107 |
| *rps11* | AB012087 | 40S ribosomal protein S11 | ribosome | AAGGAGAAGCTCCCACGTTA  TGCCAGCAATAGCCTCTCT | (Reference gene) | 73 |
| *tcra* | EU025122 | T cell antigen receptor alpha chain C region 1 | T-cells | AACCACCAGCCTTTTACAAATTCA  AGTTTTCTGATCCACTAAAGCTGA | 96.20 | 153 |
| *top2a* | XM_042735154 | DNA topoisomerase II alpha | proliferating B cells | ACTCAGCAAATGTGGGTGTTTGAT, GGTTAACATTGACTCGGAGAATAA | 99.96 | 170 |
| *vig1* | EX880905 | Viperin | antiviral | CGGGACTATAAGGTGGCTTT  AAGACCTTCCAGCGCACT | 89.60 | 110 |
| *xbp1* | XM_042753507 | X-box binding protein 1 | plasma cells | TTGGAGTTGGAGCTCGAGAATC,  GACTGGGGTTAGATACCCTGG | 93.73 | 121 |
| *zap70* | Scaf 2523 and 63374 | Zeta-chain-associated protein kinase 70 | T and NK cells | GGAACAAGCCATCATTAGCC  GTCGTCTCTCACCCTCCTG | 93.20 | 161 |

**Supplementary Fig. 1** Gill expression profiling of inflammatory, innate immune, and lymphocyte markers following CEV infection (G and H). Immunized and salt bath-rescued koi carp (Immunized) or non-immunized non-treated (Naïve) fish were infected with CEV. Prior to (0dpi) or up to 6 days dpi, we biopsied gills to profile expression of type I IFN-stimulated genes (*mx2* and *vig1*), complement component *c7a*, inflammatory markers (*il1b*, *il6a*, and *il8*), the regulatory cytokine *il10*, T cell markers (*gzma, zap70*, *cd4*, *cd8b1*, and *trca*), and neutrophil *mpo*. We selected also a panel of common carp orthologues of markers initially identified by single-cell RNA sequencing of grass carp head kidney IgM^+^ B cells. n = 4 fish per group. * indicates the statistically significant difference (at p≤0.05) between groups at ach timepoint, # indicates the statistically significant difference (at p≤0.05) between 0dpi and other time points.


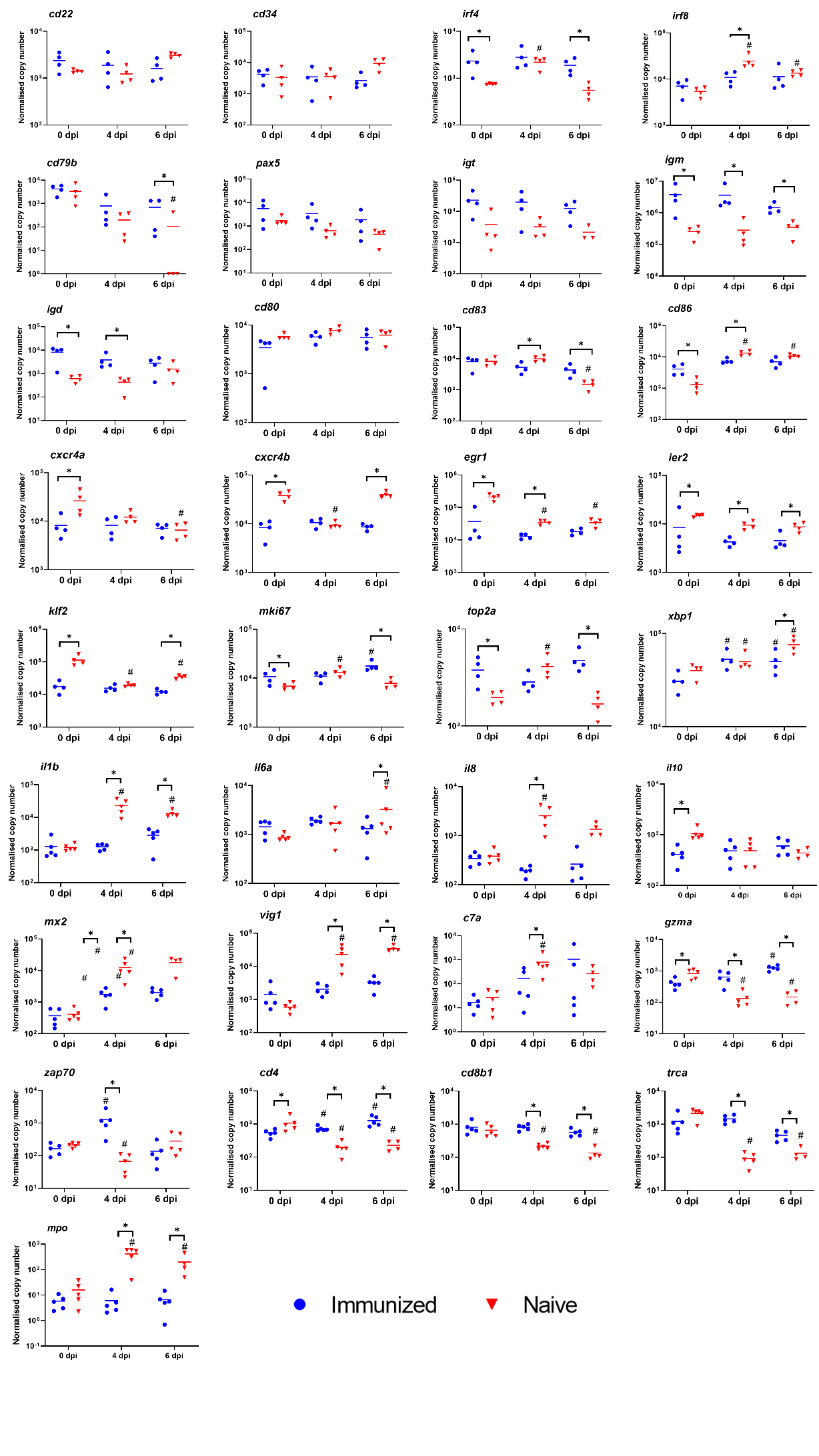

Supplement: Supplementary file 1 — Figure S1: Gill expression profiling of inflammatory, innate immune, and lymphocyte markers following CEV infection (G and H). Immunized and salt bath‐rescued koi carp (Immunized) or non‐immunized non‐treated (Naïve) fish were infected with CEV. Prior to (0dpi) or up to 6 days dpi, we biopsied gills to profile expression of type I IFN‐stimulated genes (mx2 and vig1), complement component c7a, inflammatory markers (il1b, il6a, and il8), the regulatory cytokine il10, T cell markers (gzma, zap70, cd4, cd8b1, and trca), and neutrophil mpo. We selected also a panel of common carp orthologues of markers initially identified by single‐cell RNA sequencing of grass carp head kidney IgM+ B cells. n = 4 fish per group. * indicates the statistically significant difference (at p ≤ 0.05) between groups at ach timepoint, # indicates the statistically significant difference (at p ≤ 0.05) between 0dpi and other time points. Table S1: Primers used in this study. [file JFD-49-e70185-s001.docx]
